# Metagenomic identification of pathogens and antimicrobial-resistant genes in bacterial positive blood cultures by nanopore sequencing

**DOI:** 10.3389/fcimb.2023.1283094

**Published:** 2023-12-12

**Authors:** Yahui Liu, Yumei Xu, Xinyu Xu, Xianghui Chen, Hongli Chen, Jingjing Zhang, Jiayu Ma, Wenrui Zhang, Rong Zhang, Jie Chen

**Affiliations:** ^1^ Department of Laboratory Medicine, Shanghai Xuhui District Central Hospital & Fudan University Affiliated Xuhui Hospital, Shanghai, China; ^2^ Department of Laboratory Medicine, Shanghai Post and Telecommunication Hospital, Shanghai, China; ^3^ Shanghai Diabetes Institute, Shanghai Sixth People’s Hospital Affiliated to Shanghai Jiao Tong University School of Medicine, Shanghai, China; ^4^ Precision Medicine Center, Shanghai Sixth People’s Hospital Affiliated to Shanghai Jiao Tong University School of Medicine, Shanghai, China

**Keywords:** metagenomic identification, nanopore sequencing, bacterial bloodstream infection, blood culture, antimicrobial resistance gene

## Abstract

Nanopore sequencing workflows have attracted increasing attention owing to their fast, real-time, and convenient portability. Positive blood culture samples were collected from patients with bacterial bloodstream infection and tested by nanopore sequencing. This study compared the sequencing results for pathogen taxonomic profiling and antimicrobial resistance genes to those of species identification and phenotypic drug susceptibility using traditional microbiology testing. A total of 37 bacterial positive blood culture results of strain genotyping by nanopore sequencing were consistent with those of mass spectrometry. Among them, one mixed infection of bacteria and fungi was identified using nanopore sequencing and confirmatory quantitative polymerase chain reaction. The amount of sequencing data was 21.89 ± 8.46 MB for species identification, and 1.0 MB microbial strain data enabled accurate determination. Data volumes greater than or equal to 94.6 MB nearly covered all the antimicrobial resistance genes of the bacteria in our study. In addition, the results of the antimicrobial resistance genes were compared with those of phenotypic drug susceptibility testing for *Escherichia coli*, *Klebsiella pneumoniae*, and *Staphylococcus aureus*. Therefore, the nanopore sequencing platform for rapid identification of causing pathogens and relevant antimicrobial resistance genes complementary to conventional blood culture outcomes may optimize antimicrobial stewardship management for patients with bacterial bloodstream infection.

## Introduction

Bacterial bloodstream infections (BSIs) ranging from transient bacteremia, organ infection to sepsis, and multiple organ dysfunction syndrome are major causes of infectious disease morbidity and mortality worldwide (Rutanga et al., 2018; Kern and Rieg, 2020). The increased incidence of BSI has emerged as one of the leading causes of death (Goto and Al-Hasan, 2013), especially in older and critical patients (GBD 2016 Causes of Death Collaborators, 2017). Several studies have reported that insufficient or inappropriate antibiotic treatment increases BSI-related mortality (Ibrahim et al., 2000; Kumar et al., 2009; Opota et al., 2015).

Positive blood cultures are considered the gold standard for diagnosing BSI and are commonly used in clinical microbiology practice. However, their major drawbacks include the long turnaround time and delay or failure to detect causal pathogens in patients with BSI who have received antibiotics treatment previously (Fenollar and Raoult, 2007). Positive blood culture samples usually require 1 and 2 days for the identification of bacterial species and phenotypic testing for drug sensitivity patterns, respectively, in a clinical microbiology workflow. This often results in delayed or inaccurate diagnosis of bacteremia, which ultimately leads to prolonged hospital stay, excessive costs and higher mortality rates (Dubourg and Raoult, 2016; Evans et al., 2021).

Over the last decade, advances in molecular-based methods have been introduced for the rapid identification of pathogens and antimicrobial resistance (AMR) genes in blood and blood cultures (Li et al., 2017; Sinha et al., 2018; Govender et al., 2021). Nanopore sequencing technology greatly reduces the time required for sequencing and meets the DNA sequencing requirements under several conditions because of its convenient portability. Advancements in nanopore sequencing technology have made conducting outbreak investigations on a wide range of infectious pathogens and detect AMR possible (Li et al., 2021). Several studies have revealed that real-time nanopore technology has the potential to accelerate the detection of relevant pathogens and AMR-encoding genes in positive blood cultures (Ashikawa et al., 2018; Taxt et al., 2020; Zhou et al., 2021). Accordingly, we sought to determine the performance of the nanopore sequencing assay by comparing it with matrix-assisted laser desorption/ionization time-of-flight (MALDI-TOF) mass spectrometry (MS) for pathogen identification and compare the AMR gene profiles and phenotypic drug resistance data to identify resistance mutations associated with drug resistance in positive bacterial blood culture samples.

## Methods

### Patient selection and clinical data collection

Between October 2022 and February 2023, we enrolled 37 patients who were hospitalized at Shanghai Xuhui District Central Hospital and fulfilled the diagnostic criteria for BSI with one or more positive blood culture bottles for bacteria unrelated to infection at any other site. Eligible patients were adults (age ≥18 years) who had positive blood cultures processed in the clinical microbiology laboratory using the Becton Dickinson BACTEC FX system. Patients were excluded if they had a positive blood culture in the prior week, or had a negative Gram stain or the most “common” human skin bacterial residents (Corynebacterium, Propionibacterium, Micrococcus and Brevibacterium) from positive blood culture bottles. The residual blood culture samples were collected for nanopore sequencing. On the day of blood culture collection, data from 37 routine blood examination, 37 C-reaction protein, 34 procalcitonin, 10 (1,3) β-D-glucan testing, and 10 galactomannan antigen testing were recorded as directed by the ordering physician. Gram staining and bacterial species identification using Bruker Microflex MALDI-TOF MS were performed on all 37 positive blood culture specimens. In total, 32 of the 37 specimens were tested for drug sensitivity because two patients died during hospitalization, two patients were discharged from the hospital, and one patient was considered to have a falsely negative infection, with only one blood culture identified as coagulase-negative Gram-positive cocci.

### Host-depleted DNA extraction from positive blood culture samples

To achieve better bacteria genome sample coverage (i.e., faster running time) (Charalampous et al., 2019), we adopted a host-depleted DNA extraction method, efficiently depleting human host DNA and yielding enriched bacterial DNA. First, 1 ml of positive blood culture sample was collected in a sterile 1.5 ml Eppendorf tube and centrifuged at 10000 g for 5 min to remove the supernatant. The precipitate was suspended in 1 ml normal saline for DNA extraction using the QIAamp DNA Microbiome Kit (Qiagen, Germany) according to the manufacturer’s instructions. All 37 samples were stored at 4°C for no more than 24 h before DNA extraction. Eluates were stored at -20°C until library preparation.

### Library preparation and nanopore sequencing

DNA purity was measured using a Nanodrop 1000 Spectrophotometer (Thermo Fisher Scientific, Waltham, MA, USA) and DNA concentration was measured using a Qubit 4.0 fluorometer (Thermo Fisher Scientific, Waltham, MA, USA) according to the manufacturer’s instructions. The measurements were used to obtain pure DNA and calculate the concentrations required for library preparation. Library preparation and nanopore sequencing were carried out with Rapid sequencing DNA - PCR Barcoding kit SQK-RPB004 (Oxford Nanopore Technologies, OX, UK). Library preparation included DNA tagmentation, PCR, and adapter ligation. 1–5 ng (3 µl) of high molecular weight genomic DNA and 1 µl Fragmentation Mix were mixed in a PCR tube. Tagmented DNA was obtained by incubating the tube for 1 min at 30°C and then 1 min at 80°C in a thermal cycler. Then, the PCR reaction was set up as following: 4 µl tagmented DNA, 1 µl of Rapid Barcode Primer 1–12, 25 µl LongAmp Taq 2X master mix and 20 µl Nuclease-free water to a total volume of 50 µl. The PCR protocol included initial denaturation at 95°C for 3 min, followed by 14 cycles of denaturation (95°C for 15 s), annealing (56°C for 30 s) and extension (65°C for 6 min), and final extension was at 65°C for 6 min. PCR products were subjected to a 0.6 × AMPure XP bead wash and eluted in 10 µl buffer (50mM NaCl, 10mM Tris.HCl pH 8.0). DNA concentration was measured using a Qubit 4.0 Fluorometer. Pool six to twelve barcoded libraries in the desired ratios to a total of 50–100 fmoles with 1µl Rapid Adapter. The reaction mixture was incubated for 5 min at room temperature (~ 25°C). Sequencing was performed on the GrinION platform using a SpotON Flow Cell, and ONT MinKNOW software (version 22.10.7) was used to collect the raw sequencing data. Sequencing data were collected at 30 min, and 1, 2, 4, and 24 h on the GridION platform according to the barcode of the sample number. All sequencing reads with a quality score >10 were used to form a FASTQ pass file and undergo further analysis.

### Quantitative PCR assays

qPCR was performed using TaqMan probes from Applied Biosystems to simultaneously detect and quantify pathogens and human DNA. qPCR analysis was conducted using a QuantStudio™ 7 Flex Real-Time PCR System (Applied Biosystems, USA). Human probe RPPH1 (Hs04930436_g1), *Pseudomonas aeruginosa* probe (Ba04932081_s1), and *Candida parapsilosis* probe (Fn04646221_s1) were used. For all probe-based qPCRs, the master mix consisted of 10 µl of 2 × TaqMan Universal Master Mix II with UNG (uracil-N-glycosylase, 4440038), 1 µl probe, 1 µl DNA template (1–10 ng/µl), and nuclease-free water were added to a total of 20 µl reaction system. The prepared 20 µl reactions were incubated with UNG at 50°C for 2 min. qPCR was performed as follows: 10 min at 95°C for initial denaturation, followed by 40 cycles at 95°C for 15 s and at 60°C for 60 s.

### Metagenomic data for microbial taxonomic analysis and AMR gene detection by CLC Genomics Workbench 23.0.1

The reference human database was downloaded from the ENSEMBLE Human Reference Genome GRCh37/hg19 (https://ftp.ensembl.org/pub/grch37/release-109/fasta/homo_sapiens/dna/). The microbial genome database consisted of 2897 bacteria (including 229 mycobacteria) and 440 fungi based on the list of strains for the Bruker MALDI-TOF MS instrument used in our laboratory were downloaded from National Center for Biotechnology Information (https://ftp.ncbi.nlm.nih.gov/genomes/refseq/bacteria/ and https://ftp.ncbi.nlm.nih.gov/genomes/refseq/fungi/ 03/24/2023). The drug resistance database was provided by QIAGEN Microbial Insight - Antimicrobial Resistance Version 6 (2021 - 08). This database contains peptide markers derived from the following source databases: CARD, v3.1.3, 2021 - 07 - 05 (https://card.mcmaster.ca/); ARG-ANNOT, v6, July 2019 (https://www.mediterranee-infection.com/arg-annot/); NCBI Bacterial Antimicrobial Resistance Reference Gene Database, v 3.10, 2021 – 06 – 01 (https://ftp.ncbi.nlm.nih.gov/pathogen/Antimicrobial_resistance/AMRFinderPlus/database/3.10/); ResFinder, revision eec8752, 2021 - 06 - 12 (https://bitbucket.org/genomicepidemiology/resfinder_db/src/master/). These three databases were uploaded to the CLC Genomics Workbench 23.0.1 to build a local database. Taxonomic analysis of the bacterial and fungal species was performed through the default “Data QC and Taxonomic Profiling” program by combining the reference human database and microbial genome database. The program “Data QC and Taxonomic Profiling” was used with default settings. This program was composed of three steps: (a) QC for sequencing reads (b) reads trimming and (c) host sequence removing and taxonomy profiling. AMR gene detection was carried out using the default “*De Novo* Assemble Long Reads (beta)” and “Find Resistance with Nucleotide DB” programs through the drug resistance database. The program “Find Resistance with Nucleotide DB” was defined as the percentage for minimum identity greater than 98% and the minimum length greater than 60%.

### Statistical analysis

All the statistical analyses were performed using SPSS version 25 software (IBM SPSS Statistics, USA). Quantitative data are presented as means ± standard deviation. Categorical variables are presented as absolute values and percentages. We examined the non-linear relationship between the nanopore sequencing data size and AMR gene coverage using a generalized additive model based on smooth curve fitting. When nonlinearity was detected, a recursive algorithm was used to calculate the significant inflection points in the relationship. These analyses were performed using R version 4.4.0 (R Foundation for Statistical Computing, Austria). The relationship between AMR gene coverage and sequencing data volume was calculated using the Pearson correlation coefficient with two-tailed analysis; *P* values < 0.05 were evaluated as statistically significant.

## Results

### Patient clinical characteristics and study design

Thirty-seven positive bacterial blood cultures from 37 patients were enrolled in this retrospective study. The patient demographics and clinical characteristics are shown in **Table 1**.

**Table 1:** Demographic and clinical characteristics of the 37 patients with bacterial bloodstream infection.

Characteristic	Bacterial BSI, n=37
Age, year	71.19 ± 17.85
Male, n (%)	24 (64.9%)
Laboratory results	
White blood cell, ×10^9^/L	11.98 ± 7.65
Neutrophils (%)	87.06 ± 6.62
CRP, mg/L	112.25 ± 72.97
PCT, ng/ml	11.68 ± 18.55
G test, pg/ml	61.05 ± 84.92
GM test, µg/L	0.13 ± 0.03
Outcome, n (%)	
Recovery	27 (73.0%)
Stable disease	1 (2.7%)
Progress	2 (5.4%)
Death	7 (18.9%)

CRP, C-reactive protein, BSI: bloodstream infection; PCT, procalcitonin; G test, β-D-glucan test; GM test, galactomannan antigen test.

To verify the accuracy of the nanopore sequencing data, we compared the 37 sequenced results with those of the bacterial species identification confirmed by MALDI-TOF. Non-concordant or undetectable results were verified using qPCR. Data volumes were recorded at the first time point for microbial taxonomic classification (30min or 1 h).

We analyzed the relationship between the nanopore sequencing data volume and the AMR gene coverage. Using the list of AMR genes collected at 24 h as a reference, those obtained at 2 and 4 h were compared for divergence.

Owing to the limited sample size, the number of samples for each microbial strain was relatively small. The most frequently encountered pathogens, such as Gram-negative *Escherichia coli* and *Klebsiella pneumoniae* and Gram-positive *Staphylococcus aureus* were selected for comparison between AMR genes obtained from sequencing outcomes and phenotypic antimicrobial susceptibility reports.

### Microbial species identification and data volume collection using raw nanopore sequencing readings

According to the default settings, the real-time base-calling module from the Oxford Nanopore GridION platform was used for generating 4,000 sequences per output file. The first time point for the sequencing file(s) was obtained at 30 min or 1 h in 37 bacterial positive blood culture samples, and the number of sequenced reads ranged from 4000 to 12000.

The nanopore sequencing results of the metagenomic bacterial species identification were consistent with those of the MALDI-TOF MS colonies in clinical microbiology tests. The data sizes of the 37 sequenced samples and the corresponding microbial species were 21.89 ± 8.46 MB and 15.04 ± 7.07 MB, respectively. The minimum data size of the microbial strain was 1.0 MB, which was sufficient to accurately identify the bacterial species in sample S37 (**Table 2**). In addition, sample S21, which was identified as *P. aeruginosa* by routine MALDI-TOF analysis, simultaneously mapped to two species, *P. aeruginosa* and *C. parapsilosis*, in the raw nanopore sequencing data (**Table 2**). This outcome was validated by using a different method (qPCR).

**Table 2:** Comparison of metagenomic nanopore sequencing and routine blood culture results with the corresponding data sizes of total and microbial strain in 37 bacterial positive blood culture samples.

Sample ID	Pathogen cultured by routine microbiology	Pathogen identified from metagenomic nanopore sequencing	Total data size, MB	Total reads	Microbial strain data size, MB	Microbial strain reads
S1	*Klebsiella pneumoniae*	*Klebsiella pneumoniae*	32.4	8000	25.3	6244
S2	*staphylococcus haemolyticus*	*staphylococcus haemolyticus*	30.0	8000	23.6	6298
S3	*Klebsiella pneumoniae*	*Klebsiella pneumoniae*	30.4	8000	24.9	6546
S4	*Klebsiella pneumoniae*	*Klebsiella pneumoniae*	31.7	8000	26.2	6614
S5	*Klebsiella pneumoniae*	*Klebsiella pneumoniae*	17.4	4000	14.4	3302
S6	*Enterococcus faecalis*	*Enterococcus faecalis*	15.1	4000	12.8	3393
S7	*Escherichia coli*	*Escherichia coli*	26.2	8000	17.6	5388
S8	*Staphylococcus aureus*	*Staphylococcus aureus*	15.5	4000	12.9	3325
S9	*Escherichia coli*	*Escherichia coli*	14.6	4000	12.4	3406
S10	*Staphylococcus aureus*	*Staphylococcus aureus*	25.5	8000	21.0	6577
S11	*Proteus mirabilis*	*Proteus mirabilis*	25.0	8000	20.9	6676
S12	*Klebsiella pneumoniae*	*Klebsiella pneumoniae*	17.8	4000	15.1	3402
S13	*Pseudomonas aeruginosa*	*Pseudomonas aeruginosa*	16.8	4000	12.8	3047
S14	*Escherichia coli*	*Escherichia coli*	18.5	4000	14.7	3180
S15	*Staphylococcus aureus*	*Staphylococcus aureus*	30.1	8000	24.9	6618
S16	*Pseudomonas aeruginosa*	*Pseudomonas aeruginosa*	13.4	4000	11.5	3430
S17	*Enterococcus faecalis*	*Enterococcus faecalis*	13.1	4000	10.2	3106
S18	*Klebsiella pneumoniae*	*Klebsiella pneumoniae*	*35.0*	*8000*	*24.2*	*5525*
S19	*Klebsiella pneumoniae*	*Klebsiella pneumoniae*	*18.6*	*4000*	*13.9*	*2995*
S20	*Escherichia coli*	*Escherichia coli*	13.9	4000	10.7	3090
S21	*Pseudomonas aeruginosa*	*Pseudomonas aeruginosa*	15.9	4000	4.4	1096
		*Candida parapsilosis*			9.9	2499
S22	*Aeromonas hydrophila*	*Aeromonas hydrophila*	14.4	4000	1.9	516
S23	*Escherichia coli*	*Escherichia coli*	12.3	4000	9.8	3173
S24	*Escherichia coli*	*Escherichia coli*	13.7	4000	10.4	3035
S25	*Serratia grimessi*	*Serratia grimessi*	*35.7*	*8000*	*31.7*	*7105*
S26	*Escherichia coli*	*Escherichia coli*	15.0	4000	11.1	2953
S27	*Klebsiella pneumoniae*	*Klebsiella pneumoniae*	14.2	4000	10.9	3067
S28	*Escherichia coli*	*Escherichia coli*	15.0	4000	4.4	1180
S29	*Escherichia coli*	*Escherichia coli*	*17.6*	*4000*	*14.3*	*3245*
S30	*Escherichia coli*	*Escherichia coli*	*31.6*	*8000*	*16.7*	*4232*
S31	*Klebsiella pneumoniae*	*Klebsiella pneumoniae*	13.6	4000	10.4	3049
S32	*Pseudomonas aeruginosa*	*Pseudomonas aeruginosa*	*30.3*	*8000*	*23.3*	*6157*
S33	*Streptococcus constellatus*	*Streptococcus constellatus*	10.5	4000	5.5	2077
S34	*Staphylococcus hominis*	*Staphylococcus hominis*	27.3	8000	9.1	2671
S35	*Enterococcus casseliflavus*	*Enterococcus casseliflavus*	39.5	12000	5.8	1747
	*Enterococcus gallinarum*	*Enterococcus gallinarum*			9.6	2917
S36	*Staphylococcus capitis*	*Staphylococcus capitis*	34.8	12000	16.4	5639
S37	*Enterococcus faecium*	*Enterococcus faecium*	27.5	8000	1.0	289
mean ± SD			21.89 ± 8.46		15.04 ± 7.07	

Data in italics were collected at 1 h and the other data were collected at 30 min after the start of nanopore sequencing.

### Microbial species validation by using qPCR

**Figure 1:**
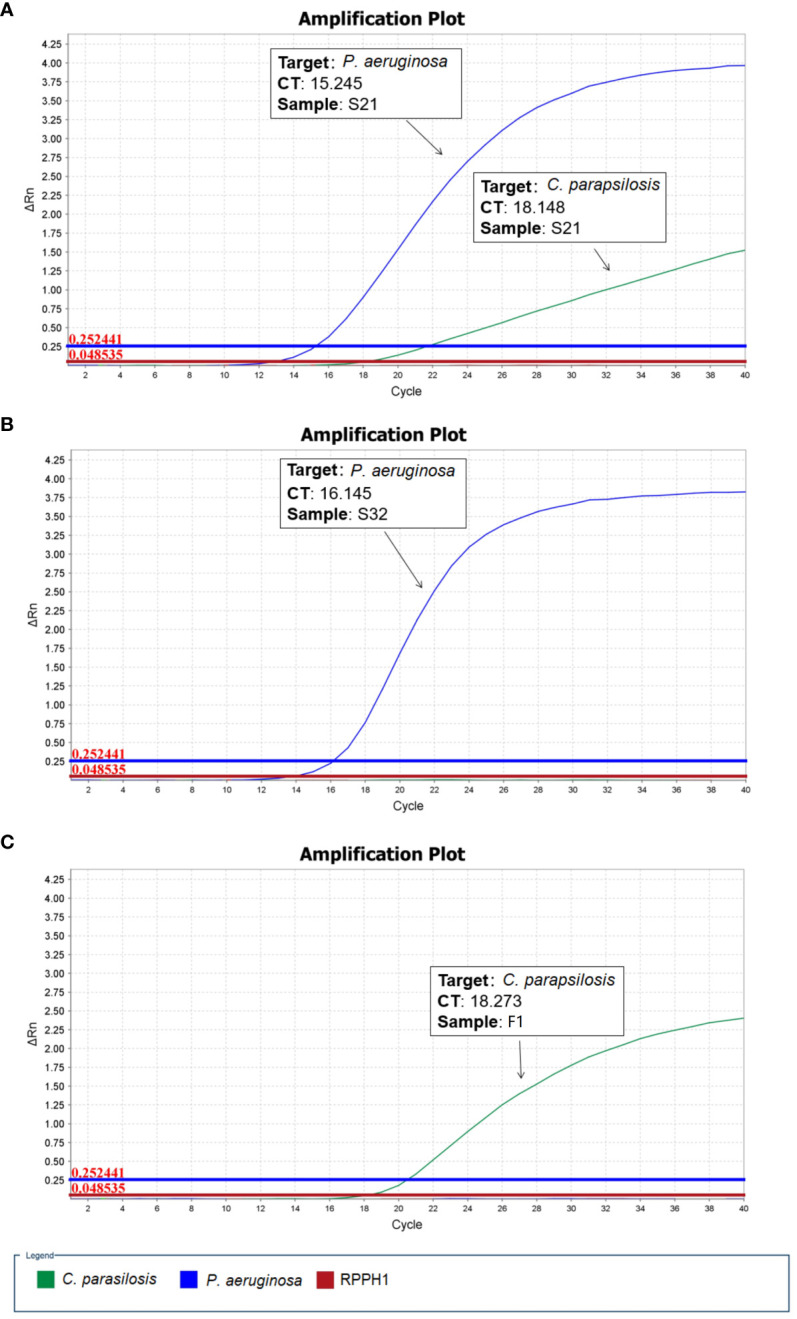
Quantitative polymerase chain reaction (qPCR) validation performed for microbial positive blood culture samples. **(A)** S21: *P. aeruginosa* and *C. parapsilosis* **(B)** S32: *P. aeruginosa* **(C)** F1: *C. parapsilosis* (RPPH1 - human probe).

As described above, two microbial species (*P. aeruginosa* and *C. parapsilosis*) were identified in sample S21 from the raw nanopore sequencing data; however, only one microbial species (*P. aeruginosa*) was obtained from blood culture by routine microbiological tests. The β-D-glucan test result for the patient was 128.01 pg/ml, which was apparently higher than the upper reference limit of 60 pg/ml. Therefore, one fungal blood culture-positive specimen, identified as *Candida parapsilosis* (F1), was collected. To validate the reliability of the undetected *C. parapsilosis*, sample S32 and a fungal-positive sample F1, identified as *P. aeruginosa* and *C. parapsilosis* respectively by routine microbiological tests, were used as controls for qPCR. As shown in **Figure 1**, the qPCR results are in good agreement with the nanopore sequencing taxonomic profiles for S21, S32 and F1. All the samples were subjected to host-depleted DNA extraction, and human DNA that was intrinsically abundant in the blood culture samples was adequately eliminated.

### The relationship between nanopore sequencing data size and AMR gene coverage

**Figure 2:**
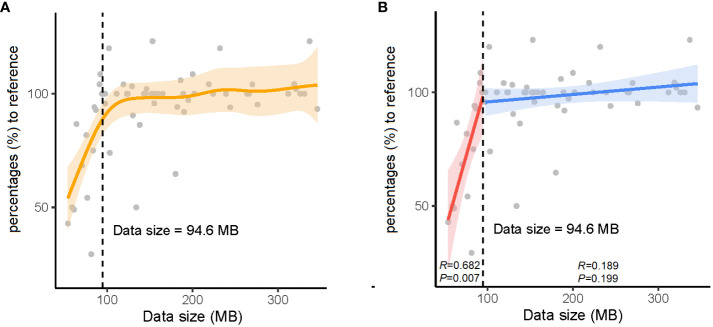
The relationship between data sizes and percentages (%) to the reference antimicrobial resistance (AMR) genes. **(A)** The orange solid line denotes the non-linear trend fitted by the generalized additive model (GAM). The vertical black dashed line and inset number represent the identified nanopore sequencing data size threshold. **(B)** The blue and red solid lines indicate the linear fits on either side of the nanopore sequencing data size threshold. The shaded area indicates the 95% confidence interval of the regression lines.

As real-time sequencing and analysis have been widely used in nanopore sequencing, we collected data at three time points (2, 4, and 24 h) for AMR gene analysis in 32 positive blood culture samples. The number of AMR genes obtained at 24 h was used as a reference, and those obtained at 2 h and 4 h were collected by calculating the percentage relative to the reference (**Table 3**). We subsequently calculated the significant inflection point (data size = 94.6 MB) for the fitted curve by generalized additive model between the nanopore sequencing data size and the corresponding percentages (%) to the reference (**Figure 2**). The relationship between the nanopore sequencing data size and the percentages (%) to the reference AMR genes is shown in **Figure 2**. Sequencing data sizes positively correlated to the percentages (%) to reference AMR genes when the sequencing data sizes were less than 94.6 MB. (Pearson correlation coefficient = 0.682; *P* = 0.007) (**Figure 2B**). The correlation between sequencing data sizes and the percentages to reference AMR genes was not statistically significant, as the sequencing data sizes were greater than or equal to 94.6 MB. (Pearson correlation coefficient = 0.189; *P* = 0.199) (**Figure 2B**). Therefore, nearly all the AMR genes were detected with a sequencing data volume greater than or equal to 94.6 MB for bacterial AMR gene profiles.

**Table 3:** Association of the data sizes, number of AMR genes, and percentages (%) with the reference at 2, 4, and 24 h in 32 bacterial positive blood culture samples.

Sample ID	2 h			4 h			24 h		
	Data size, MB	Number of AMR genes	% to the reference	Data size, MB	Number of AMR genes	% to the reference	Data size, MB	Number of AMR genes	% to the reference
S1	130.0	19	90.5	276.0	20	95.2	1393.0	21	100.0
S2	165.0	15	100.0	346.0	14	93.3	1772.0	15	100.0
S3	153.0	16	123.1	337.0	16	123.1	1700.0	13	100.0
S4	143.0	23	100.0	319.0	24	104.3	1608.0	23	100.0
S5	123.0	21	100.0	264.0	21	100.0	1331.0	21	100.0
S6	124.0	11	100.0	266.0	11	100.0	1505.0	11	100.0
S7	146.0	48	102.1	321.0	48	102.1	1751.0	47	100.0
S8	111.0	16	100.0	239.0	16	100.0	1290.0	16	100.0
S9	119.0	51	104.1	270.0	51	104.1	1434.0	49	100.0
S10	155.0	15	100.0	326.0	15	100.0	1761.0	15	100.0
S11	152.0	20	100.0	331.0	20	100.0	1812.0	20	100.0
S12	90.9	25	104.2	219.0	25	104.2	1157.0	24	100.0
S13	103.0	37	74.0	244.0	47	94.0	1290.0	50	100.0
S14	94.6	25	100.0	190.0	23	92.0	1096.0	25	100.0
S15	154.0	18	100.0	312.0.	18	100.0	1679.0	18	100.0
S16	83.4	39	75.0	182.0	49	94.2	952.0	52	100.0
S17	93.8	4	100.0	202.0	4	100.0	1044.0	4	100.0
S18	70.3	13	68.4	159.0	19	100.0	805.0	19	100.0
S19	75.6	18	81.8	152.0	22	100.0	804.0	22	100.0
S20	84.9	32	94.1	186.0	36	105.9	929.0	34	100.0
S21	81.3	5	29.4	180.0	11	64.7	909.0	17	100.0
S22	59.0	1	50.0	134.0	1	50.0	731.0	2	100.0
S23	102.0	42	120.0	232.0	42	120.0	1147.0	35	100.0
S24	97.5	36	100.0	195.0	35	97.2	1014.0	36	100.0
S25	72.2	/	/	145.0	/	/	754.0	/	/
S26	91.8	38	108.6	200.0	38	108.6	998.0	35	100.0
S27	86.6	13	92.9	189.0	14	100.0	993.0	14	100.0
S28	76.6	13	54.2	155.0	23	95.8	803.0	24	100.0
S29	53.9	9	42.9	145.0	21	100.0	771.0	21	100.0
S30	64.0	26	86.7	129.0	31	103.3	735.0	30	100.0
S31	97.4	22	95.7	224.0	23	100.0	1188.0	23	100.0
S32	61.0	25	49.0	138.0	44	86.3	749.0	51	100.0

AMR, antimicrobial resistance; /, No AMR gene was mapped to the drug resistance database.

### Comparison between the AMR genes from raw nanopore sequencing data and phenotypic antimicrobial susceptibility testing

We selected blood culture samples positive for the most common bacterial species, including three *S. aureus*, ten *E. coli* and seven *K. pneumoniae* with standard phenotypic AST in our study. The phenotypic AST results for the three bacterial strains are shown in **Supplementary Table 1**.

**Figure 3:**
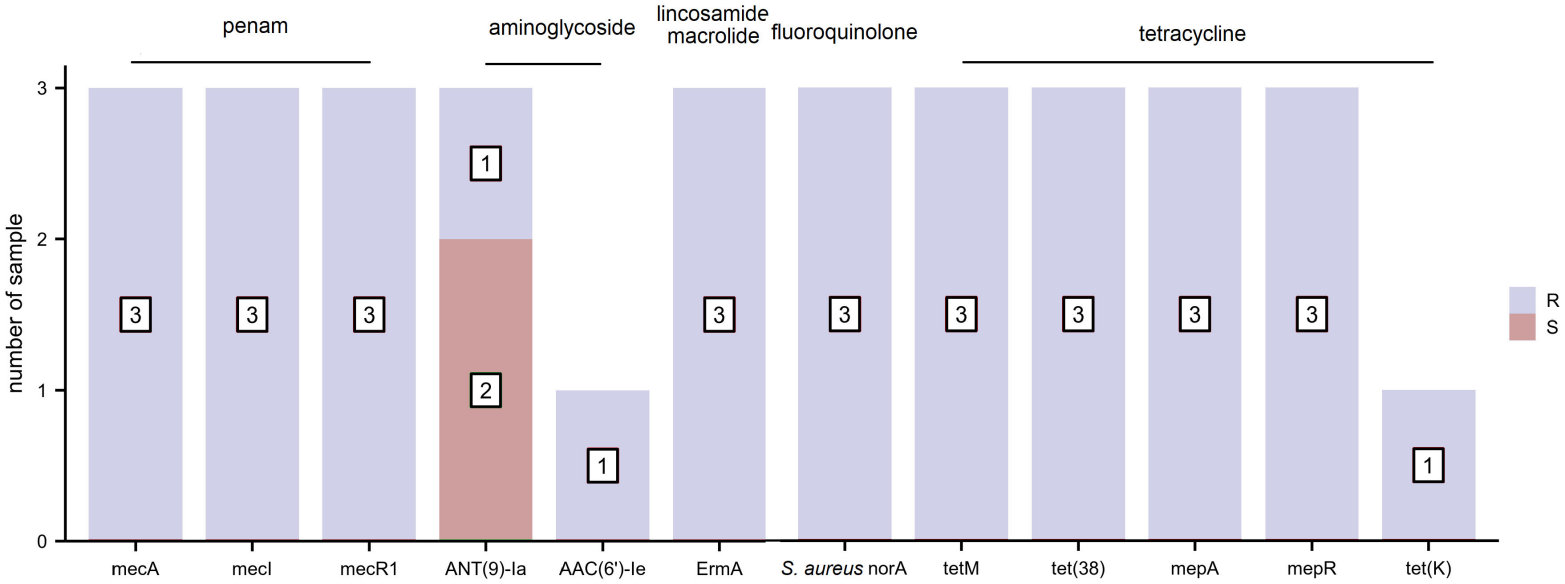
The distribution of antimicrobial resistance (AMR) genes and related phenotypic antimicrobial susceptibility testing (AST) outcomes for S. aureus (R, resistant and S, sensitive).

The distribution of AMR genes and related phenotypic AST outcomes for *S. aureus* are listed in **Figure 3**. Resistance genes, including those toward penams (*mecA*, *mecI*, and *mecR1*), aminoglycosides (*AAC(6’)-Ie-APH(2’’)-Ia*), macrolides/lincosamides (*ErmA*), fluoroquinolones (*S. aureus norA*), and tetracyclines (*tetM*, *tet(38)*, *mepA*, *mepR*, and *tet(K)*), were associated with clinically relevant resistance phenotypes. The resistance gene, *ANT(9)-Ia*, was detected in all three *S. aureus* strains regardless of whether they exhibited phenotypic resistance or sensitivity to aminoglycoside antibiotics. None of the AMR genes harbored a clinically sensitive phenotype.

**Figure 4:**
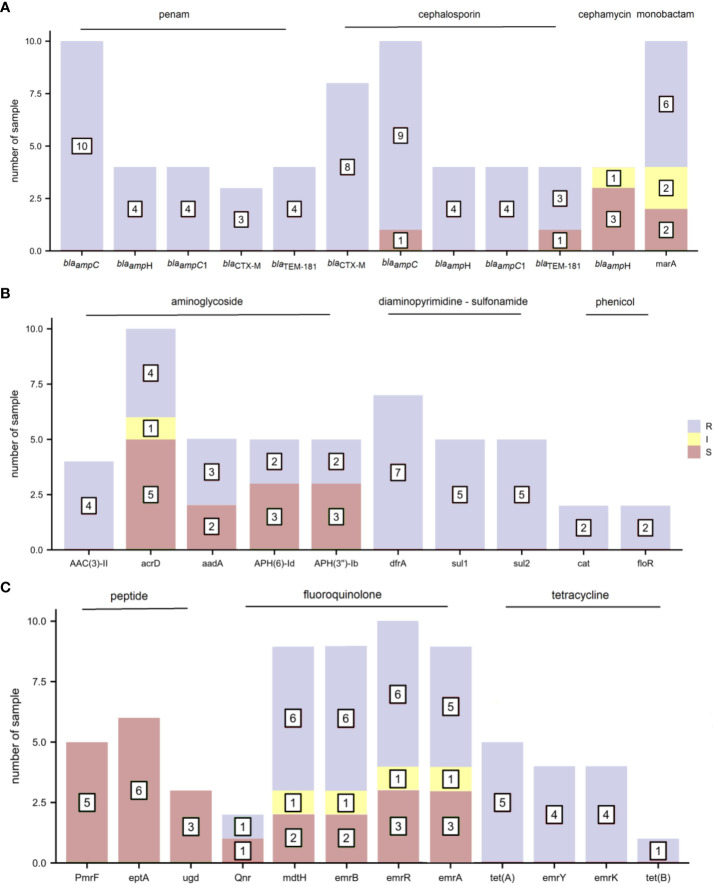
The distribution of antimicrobial resistance (AMR) genes and related phenotypic antimicrobial susceptibility testing (AST) outcomes for *E. coli* (R, resistant; I, intermediate; and S, sensitive). **(A)** penam, cephalosporin, cephamycin, and monobactam **(B)** aminoglycoside, diaminopyrimidine-sulfonamide, and phenicol **(C)** peptide, fluoroquinolone, and tetracycline.

The distribution of AMR genes and related phenotypic AST outcomes in *E. coli* are shown in **Figure 4**. As several antibiotics are sometimes attributed to one type of drug class, AMR genes presenting phenotypic resistance to any antibiotic were defined as resistance to that type of drug class. The resistance gene *bla*_ampC_ was associated with ampicillin resistance. The resistance gene *bla*_CTX-M_ corresponded to cefuroxime, ceftriaxone, and cefepime resistance and cefepime intermediary. The resistance genes *AAC(3)-IId* and *AAC(3)-IIe* were detected in the four gentamicin-resistant samples. Trimethoprim-sulfamethoxazole resistance was observed in seven samples containing the diaminopyrimidine-sulfonamide resistance genes *dfrA17*/*dfrA14*/*dfrA12*, *sul1* and *sul2*. The resistance genes *tet(A)*, *emrY*, *emrK*, and *tet(B)* were mapped to tetracycline resistance. The other *E. coli* drug-resistant phenotypes were not consistent with drug resistance genes.

**Figure 5:**
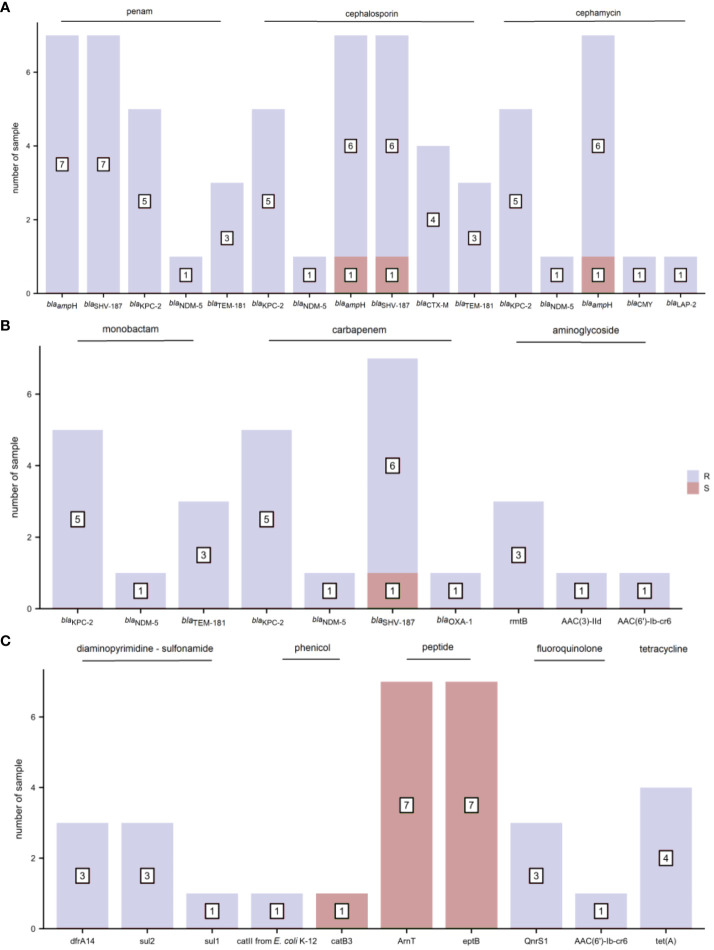
The distribution of antimicrobial resistance (AMR) genes and related phenotypic antimicrobial susceptibility testing (AST) outcomes for *K. pneumoniae* (R, resistant and S, sensitive). **(A)** penam, cephalosporin, and cephamycin **(B)** monobactam, carbapenem, and aminoglycoside **(C)** diaminopyrimidine-sulfonamide, phenicol, peptide, fluoroquinolone, and tetracycline.

The distribution of AMR genes and the associated phenotypic AST outcomes for *K. pneumoniae* are shown in **Figure 5**. Resistance genes *bla*_ampH_ and *bla*_SHV-187_ were associated with ampicillin resistance. The resistance genes *bla*_KPC-2_ and *bla*_NDM-5_ corresponded to penams (amoxicillin/clavulanic acid, ampicillin/sulbactam, and piperacillin/tazobactam), cephalosporins (cefoperazone/sulbactam, cefazolin, cefuroxime, ceftazidime, ceftriaxone, and cefepime), cephamycins (cefoxitin), monobactams (aztreonam), and carbapenems (imipenem, meropenem, and ertapenem) phenotypic resistance. In addition, *bla*_KPC-2_ demonstrated a one-to-one correspondence with the resistance phenotype of class A serine carbapenemases, and *bla*_NDM-5_ with that of class B metallo-β-lactamases. The resistance gene *rmtB* was found concurrently in tobramycin-, gentamicin-, and amikacin-resistant samples; but *AAC(3)-IId* and *AAC(6’)-Ib-cr6* were present only in gentamicin-resistant and tobramycin-resistant samples, respectively. The diaminopyrimidine-sulfonamide resistance genes *dfrA14* and *sul2* were detected in three trimethoprim-sulfamethoxazole-resistant samples. The phenicol resistance gene *catII from E. coli K-12*, but not *catB3*, was mapped to one chloramphenicol-resistant sample. The resistance gene *tet(A)* corresponded to tetracycline resistance. The other AMR genes did not precisely fit the corresponding drug-resistant phenotypes.

### Nanopore sequencing provided information concerning microbial species identification and AMR genes analysis more rapidly than conventional microbiology testing

**Figure 6:**
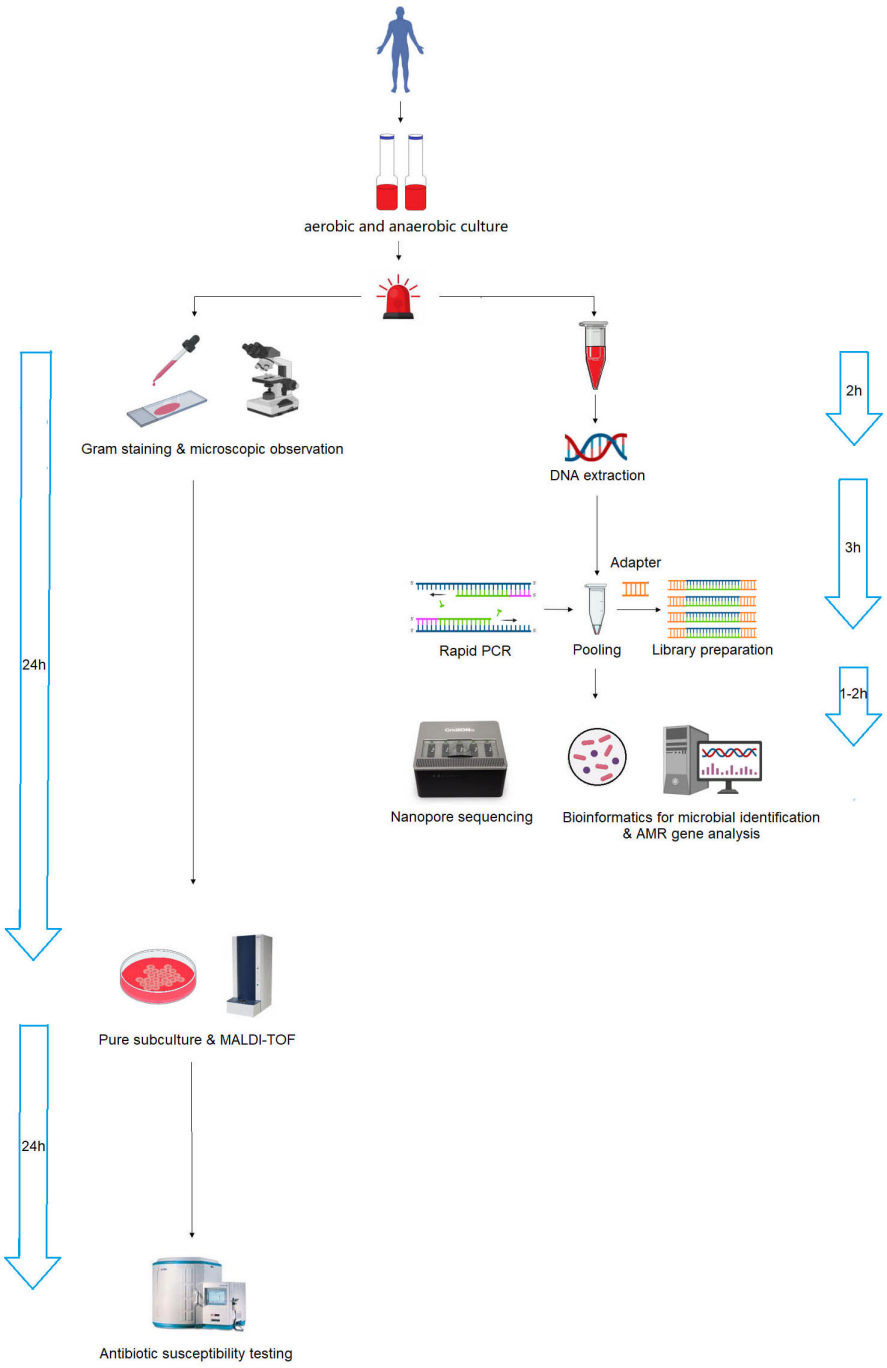
Schematic workflow of nanopore sequencing and conventional microbiology testing for positive blood cultures. Nanopore sequencing dramatically accelerated the process, reducing the turnaround time from 24–48 h to 6–7 h.

The nanopore sequencing workflow is illustrated in **Figure 6**. After the bottle flags positive, we must subculture to obtain pure colonies before we can identify by MALDI-TOF and sent to AST in a routine microbiology laboratory, whereas with the nanopore method, we can skip the subculture and go straight to extraction and library preparation. The turnaround time for microbial species identification and AMR gene analysis was 6 and 7 h, respectively, which was significantly shorter than that of conventional microbiology testing, which was 24 and 48 h after positive blood cultures were alarmed. The rapid identification of pathogens and AMR genes contributes to the early and appropriate treatment of severe bacterial BSI.

## Discussion

Early and accurate diagnosis and administration of appropriate antimicrobials are essential for improving the prognosis and reducing the mortality rate in patients with BSI (Timsit et al., 2020; Kadri et al., 2021), especially in drug-resistant bacterial infections (Xu et al., 2019). In addition, the risk of 30-day mortality gradually increased with inappropriate antimicrobial treatment at 12, 24, 48, and 72 h (Van Heuverswyn et al., 2023). We analyzed the bacterial-positive blood culture samples using a nanopore metagenomic sequencing protocol for pathogen and AMR gene identification within 6–7 h. The total assay time was considerably shorter than that of traditional microbiological tests (24–48 hours). Relatively high cost and the potential sequencing errors inherent to the nanopore platform were the challenges or limitations encountered specifically with the new sequencing method. In our study, the adoption of host-depleted DNA extraction and the reasonable assessment of nanopore sequencing data size contributed to reduce the cost for sequencing. Besides that, updating the Super-accurate basecalling algorithm in GridION and improvement of sequencing depth were conducted to ensure the accuracy and reliability of the sequencing data. Until now, several studies have reported using nanopore metagenomic or targeted sequencing for microbial species identification from blood (Ashikawa et al., 2018; Sakai et al., 2019; Hong et al., 2023). Pathogen identification and related antimicrobial drug resistance genes prediction were conducted from simulated BSI samples (Zhou et al., 2021). In this study, we presented a promising approach to identification of responsible pathogens and antimicrobial resistant genes under the real-world clinical scenarios.

The nanopore sequencing workflow included host-depleted microbial DNA extraction, library preparation, GridION sequencing, and real-time data analysis. In contrast to the standard blood culture, bacterial species identification of 37 BSI samples from raw nanopore sequencing data was in good agreement, except for sample S21, which was mapped to bacterial and fungal co-infection using the established microbial genome database. The culture-based method identified *P. aeruginosa* only in sample S21; however, qPCR validation detected characteristic amplification curves of *P. aeruginosa* and *C. parapsilosis*, consistent with the nanopore sequencing data findings. Altun et al. reported that the molecular diagnostic technique of the BioFire FilmArray platform detected additional microorganisms compared to routine blood culture in 3.6% of the samples (Altun et al., 2013). Similar to other studies, routine microbial cultures were negative, likely owing to prior antibiotic administration (Hanna-Wakim et al., 2015; Ramanan et al., 2017) or requiring more time to become positive for fungal species (Ransom et al., 2021). The average raw data size for microbial species identification was 21.89 MB for the 37 positive blood culture samples in our study, which was comparable to the data volume obtained using the shotgun metagenomics platform (Liu et al., 2021). Only 1.0 MB microbial strain data were precisely mapped at the species level in sample S37, mainly because the host-depleted DNA extraction protocol was used for microbial DNA enrichment, and exponentially growing organisms were easily detected in blood culture bottles.

To obtain the AMR gene profile information as soon as possible, real-time data analysis with nanopore sequencing reads at 2, 4, and 24 h, corresponding to the data sizes were recorded to define the minimal data quantity. A data size of 94.6 MB was considered the watershed level for nearly complete bacterial AMR gene profiles. When the volume of data was less than 94.6 MB, the coverage across drug-resistance genes was proportional to the amount of data (*P* = 0.007). The correlation disappeared when the volume of data was greater than or equal to 94.6 MB. In addition, the number of AMR genes at 2 or 4 h was higher than that at 24 h in samples S3, S7, S9, S12, S23, and S26 owing to the correction difference of an iterative algorithm at different time points.

To assess the relationship between the AMR genes and routine phenotypic AST, we selected three common bacterial species: *S. aureus*, *E. coli*, and *K. pneumoniae* for the present study. Although scientists can determine the causal relationship between genetic alterations and global phenotypic changes, the relationship is not always a simple one-to-one correspondence (Suzuki et al., 2014), and the underlying mechanisms of drug sensitivity or resistance are not always straightforward (Jing et al., 2017). We determined the correlation between the phenotype and genotype of some drug-resistant bacteria, such as *tet(A)* & tetracycline for *E. coli* and *K. pneumoniae*, e*rmA* & lincosamide and macrolide for *S. aureus*, *dfrA* & trimethoprim-sulfamethoxazole for *E. coli* and *K. pneumoniae*, *bla*_KPC-2_ & class A serine carbapenemases for *K. pneumoniae*, and *bla*_NDM-5_ & class B metallo-β-lactamases for *K. pneumoniae*. No relationship between some other AMR genes and phenotypic antimicrobial resistance was confirmed (e.g., *ANT(9)-Ia* & aminoglycoside for *S. aureus*). Many complex resistance mechanisms coupled with the fact that AMR genes may not always be expressed make the prediction of phenotypic antimicrobial resistance from genotypic AMR data challenging. Despite this, accurate identification of the relationship between phenotypic resistance and AMR gene profiles would improve antimicrobial treatment outcomes and facilitate personalized regimens for patients with bacterial infections (Boolchandani et al., 2019).

This new nanopore sequencing technology can be used complementary to conventional blood culture methods to streamline laboratory workflow, reducing the time required for the identification of BSI pathogens and relative AMR gene profiles. Finally, treatment outcomes in patients with bacterial BSI could be improved by the application of accurate pathogen identification and the rational selection of antimicrobial drugs based on rapid and novel molecular techniques.

However, this study has several limitations. First, the present findings were derived from a single-center cohort; therefore, future investigations are warranted to validate these results in a multicenter setting. Second, although only a limited number of samples and types of bacterial species were collected and included in this study, a larger sample size and a broader range of pathogens, including diverse AMR phenotypes should be assessed to understand the reliability and clinical utility of this approach. Third, the first sequencing output file comprised 4000 reads based on the default setting, and we collected data at specified time points. These could be further optimized for less reads setting or real-time signal acquisition from available software package, which could accelerate the speed for the identification of causal pathogens and AMR genes in bacterial positive blood culture samples.

Based on our findings, the results of nanopore sequencing are informative for preliminary clinical decision making due to the shorter turnaround time. As rapid sequencing from blood culture samples to detect causing pathogen and antimicrobial resistance gene profile becomes a reality, the potential for integrating nanopore sequencing with other diagnostic methods may customize treatment regimens to increase cure rates and survival rates in patients with bloodstream infections. The scalability of this method to ever larger populations and to longer timescales is a priority. Different pathogen types, optimization of the experimental method and compatibility of different blood culture systems in multicenter laboratories are all important factors to be considered. In future studies, we hope to validate our findings in large, multicenter, prospective studies.

## Data availability statement

The data presented in the study are deposited in the NODE repository, accession number OEP004528, which can be visited in https://www.biosino.org/node/project/detail/OEP004528.

## Ethics statement

The study was approved by the ethics committee of Shanghai Xuhui District Central Hospital. Written informed consent was obtained from all study patients/participants [legal guardian/next of kin].

## Author contributions

YL: Data curation, Formal Analysis, Methodology, Writing – original draft. YX: Data curation, Methodology, Writing – original draft. XX: Data curation, Methodology, Writing – original draft. XC: Data curation, Writing – original draft. HC: Data curation, Writing – original draft. JZ: Data curation, Writing – original draft. JM: Data curation, Writing – original draft. WZ: Data curation, Writing – original draft. RZ: Data curation, Methodology, Conceptualization, Writing – review & editing. JC: Conceptualization, Data curation, Methodology, Writing – review & editing.
